# An unusual complication of acute appendicitis: isolated superior mesenteric venous pylephlebitis

**DOI:** 10.1259/bjrcr.20220111

**Published:** 2023-10-18

**Authors:** Habib Bellamlih, Amine Bentahar, Khalil Chafi, Moncef salek, Mohammed Es-said Ramaraoui, Soufiane Belabbes, Brahim Zinoun, Taoufik Africha

**Affiliations:** 1 Radiology Department, Moulay Ismail Military Hospital, Meknes, Morocco; 2 Sidi Mohamed Ben Abdellah University, Fez, Morocco; 3 Surgery department, Avicenna Military Hospital of Marrakech, Marrakech, Morocco

## Abstract

Septic thrombophlebitis of the portal vein or one of its tributaries is referred to as pylephlebitis. It is unusual to have superior mesenteric venous thrombophlebitis. It frequently arises as a result of an infection in the portal venous system’s drainage area, such as appendicitis or diverticulitis.

Preoperative diagnostic imaging can help in the early diagnosis of acute phase pylephlebitis.

A case of acute appendicitis complicated by an intra-abdominal abscess and superior mesenteric venous pylephlebitis is presented. Appendicectomy, abscess drainage, and antibiotic and anticoagulant treatment resulted in a full recovery. After two months, follow-up imaging revealed that the superior mesentric vein had been completely canalised.

## Clinical presentation

A 40-year-old male arrived at the emergency department with a 5-day duration of abdominal discomfort, particularly heightened in the right lower quadrant, accompanied by fever and a sensation of weakness. There were no instances of bloody stools, feelings of nausea, vomiting, or issues related to urination.

Upon admission, his axillary temperature measured 39°C, heart rate was 88 beats per minute, blood pressure stood at 121/67 mm Hg, and his oxygen saturation level registered as 98% while breathing room air.

During the physical examination, abdominal tenderness was identified in the right lower quadrant. The examinations of other bodily systems yielded no noteworthy findings.

## Investigations

The initial laboratory assessments revealed heightened levels of white blood cells (19,300 /mm³) and an elevated C-reactive protein (245 mg l^−1^). Liver enzyme levels demonstrated increased values, with aspartate aminotransferase at 65 IU l^−1^, alanine aminotransferase at 78 IU l^−1^, gamma-glutamyltransferase at 145 IU l^−1^, and alkaline phosphatase at 132 IU l^−1^.

## Imaging findings

**Figure 1.**
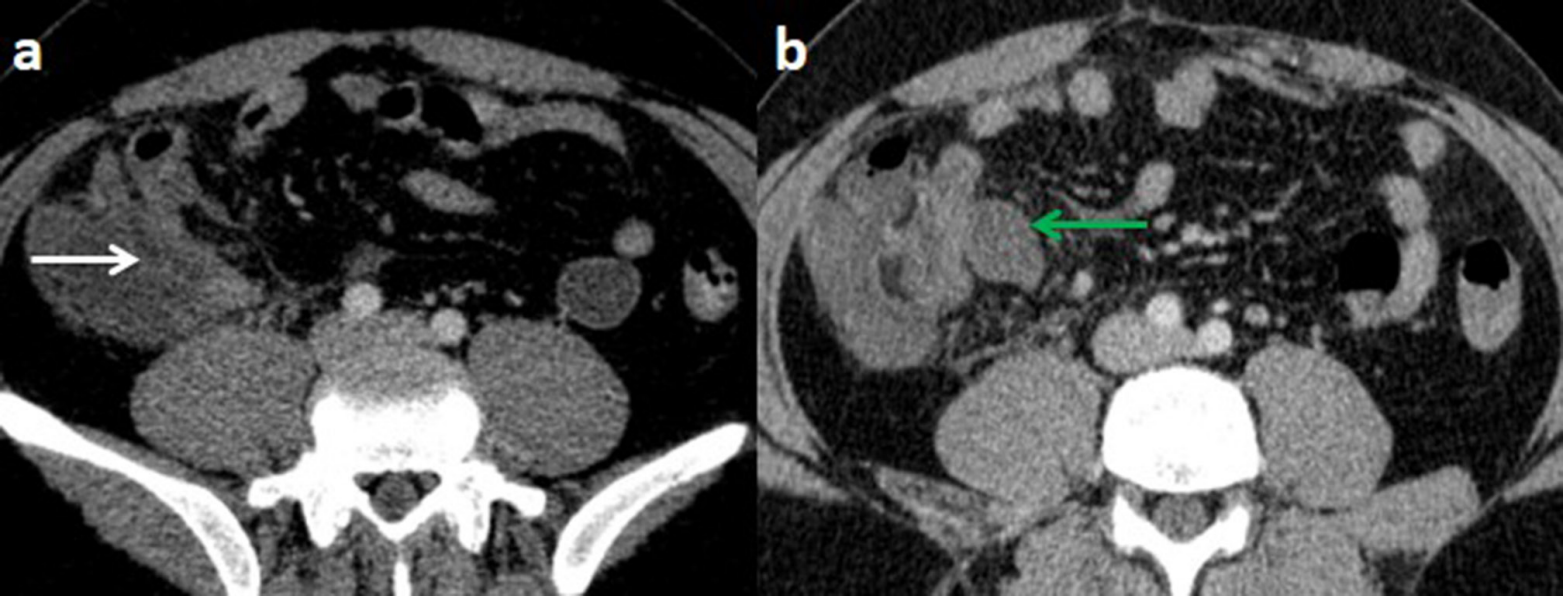
Computed tomography scan of the abdomen with intravenous contrast at portal phase (with no oral contrast) on axial plan (**a,b**) showing an enlarged and thick-walled appendix (white arrow), with a fluid collection in the latero cecal region (green arrow).

**Figure 2.**
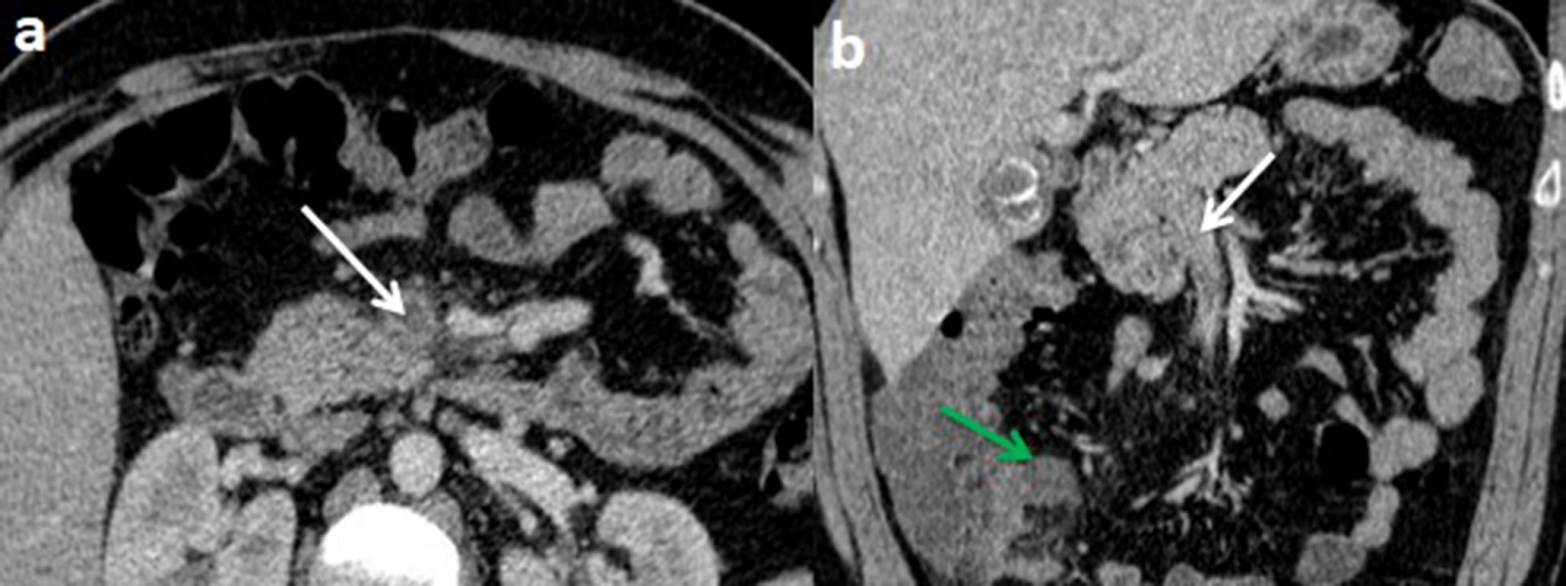
Computed tomography scan of the abdomen with intravenous contrast at portal phase (with no oral contrast) on axial plan (**a**) and coronal reconstruction demonstrating non-enhancing low-attenuation thrombi within the blood vessel lumen of the superior mesenteric vein and its branches (white arrows) with a fluid collection in the latero cecal region (green arrow).

An abdominal and pelvic contrast-enhanced computed tomography (CT) scan was conducted without oral contrast, demonstrating a dilated, hyperenhancing appendix as well as a fluid collection measuring 32 × 20 mm in the latero-caecal region with no internal gas (Figure 1). There was also a thrombus in the lumen of the superior mesenteric vein (SMV) and its branches, as well as inflammatory alterations in the surrounding fat (Figure 2).

The diagnosis of pylephlebitis was confirmed by positive blood cultures for Escherichia coli.

## Treatment, outcome and follow-up

Laparotomy confirmed a gangrenous appendicitis and an appendectomy was performed followed by drainage of the abscess. No bowel ischaemia was detected.

Post-operatively, the patient was treated with a two-week course of antibiotics and anticoagulation. Within four days, the clinical symptoms had mostly disappeared and the inflammatory markers had gradually returned to normal.

An abdominal and pelvic ultrasonography examination two months later revealed SMV recanalisation.

## Differential diagnosis

Haematological disorders (such as essential thrombocythaemia, polycythemia), hypercoagulable states (such as pregnancy, malignancy), abdominal trauma, and others are all possible causes in thrombi in the SMV.

## Discussion

Septic thrombosis of the portal vein and its branches is referred to as pylephlebitis. It typically occurs together with an abdominal infection that is suppurative. Diverticulitis and appendicitis are the two most typical intra-abdominal causes. 10% of all instances of pylephlebitis that appear are due to appendicitis.^1^ Other causes include acute cholecystitis, amoebic colitis, haemorrhoidal disease, necrotising pancreatitis, and inflammatory bowel disease.^2,3^

Pylephlebitis must be diagnosed right away because timely treatment significantly lowers morbidity and fatality rates.^4^

Unknown is the frequency of SMV thrombophlebitis. It might not be noticed during laparotomy and might be overlooked at autopsy.^5^

To distinguish pylephlebitis secondary to infections from non-infectious thrombosis, it is essential to note its typically non-occlusive nature and the absence of portal hypertension.^6^

Pylephlebitis has non-specific clinical characteristics. Fever, chills, malaise, right quadrant discomfort, and soreness are common first clinical symptoms. These symptoms are similar to those of the main illness.^7^

Blood cultures should be taken in any patient with acute portal vein thrombosis, fever, leukocytosis, and increased liver enzymes, regardless of whether an intra-abdominal infection is suspected.^8^ Blood cultures are positive in 50 to 88% of patients. Typically, laboratory testing is unspecific. The most prevalent anomalies, which were also observed in our patient, were leukocytosis, high alanine transaminase/aspartate transaminase, and bilirubin levels, and increased CRP levels.^9^

Early detection of pylephlebitis and its underlying aetiology requires imaging assessment. Although ultrasound can detect portal vein thrombosis and symptoms of the underlying abdominal inflammatory process, its accuracy can be influenced by patient compliance, intestinal gas interference, and operator skill. CT scan, on the other hand, is regarded as the ideal imaging modality for detecting pylephlebitis and any accompanying intra-abdominal disease.^10^ During CT, the presence of thrombi or air inside the portal and/or superior mesenteric venous system validates the diagnosis of pylephlebitis. CT, on the other hand, may not identify thrombi limited to smaller vessels.^4^ In addition to detecting thrombi, it is critical to assess the intrahepatic portal venous system and hepatic parenchyma.

Unopacified portal vein branches, intrahepatic abscess development, and variations in parenchymal attenuation are also related findings.^11,12^ It is important to highlight that thrombi in the portal and/or mesenteric veins can occur in a variety of circumstances.^4^

Combining the clinical history with proper imaging use and clinical correlation of results is critical in detecting pylephlebitis.

Pylephlebitis is treated by managing the main septic disease with an arsenal of broad-spectrum antibiotics and appropriate surgical surgery.^12^ Broad-spectrum antibiotics that cover a broad variety of bacteria, including Gram-negative bacilli, anaerobes, and aerobes, should be started as soon as possible and modified depending on culture findings.^12^

The total duration of antibiotic treatment for pylephlebitis has yet to be determined. However, antibiotics should be administered for at least 4 weeks to avoid the formation of hepatic abscesses, which is a frequent consequence.^13^

The use of anticoagulation in pylephlebitis has been controversial. According to some specialists, anticoagulation is utilised to prevent intestinal ischaemia and infarction induced by thrombus expansion.^14^ Despite this, there is no agreement on the best duration of anticoagulant treatment for pylephlebitis.^15^ The role of thrombolytic treatment in pylephlebitis is unknown and warrants additional research. Due to the increased risk of thrombosis recurrence, surgery on thrombosed and infected arteries is no longer commonly undertaken.^16^ However, it may still be considered in patients who do not respond to antibiotic and anticoagulation therapy.^17,18^ The decision for surgical intervention should be made on a case-by-case basis, considering individual patient factors and the severity of the condition.

Patients with pylephlebitis are at higher risk of mortality primarily because of sepsis resulting from a severe intra-abdominal infection rather than the thrombosis leading to bowel infarction.^13,16^ However, advancements in medical practices, such as early detection through imaging techniques and the use of potent antibiotic therapies, have significantly improved the survival rate, reducing mortality to 25% in recent years.^16^

## Learning points

• A uncommon but deadly complication of acute appendicitis is superior mesenteric venous thrombophlebitis.

• For detecting pylephlebitis and assessing any concomitant intra-abdominal infections, a CT scan is the recommended imaging approach.

• Addressing the core septic process is essential for effective pylephlebitis therapy. This involves using broad-spectrum antibiotics to treat the infection and, if necessary, adopting appropriate surgical procedures.
